# Absence of PTHrP Nuclear Localization and Carboxyl Terminus Sequences Leads to Abnormal Brain Development and Function

**DOI:** 10.1371/journal.pone.0041542

**Published:** 2012-07-23

**Authors:** Zhen Gu, Yahong Liu, Yongjie Zhang, Shulei Jin, Qi Chen, David Goltzman, Andrew Karaplis, Dengshun Miao

**Affiliations:** 1 State Key Laboratory of Reproductive Medicine, The Research Center for Bone and Stem Cells, Department of Anatomy, Histology and Embryology, Nanjing Medical University, Nanjing, Jiangsu, China; 2 Department of Pathophysiology, Nanjing Medical University, Nanjing, Jiangsu, China; 3 Department of Medicine, McGill University, Montreal, Canada; Max-Delbrück Center for Molecular Medicine (MDC), Germany

## Abstract

We assessed whether the nuclear localization sequences (NLS) and C terminus of parathyroid hormone-related protein (PTHrP) play critical roles in brain development and function. We used histology, immunohistochemistry, histomorphometry, Western blots and electrophysiological recordings to compare the proliferation and differentiation of neural stem cells, neuronal hippocampal synaptic transmission, and brain phenotypes including shape and structures, in *Pthrp* knock-in mice, which express PTHrP (1–84), a truncated form of the protein that is missing the NLS and the C-terminal region of the protein, and their wild-type littermates. Results showed that *Pthrp* knock-in mice display abnormal brain shape and structures; decreased neural cell proliferative capacity and increased apoptosis associated with up-regulation of cyclin dependent kinase inhibitors p16, p21, p27 and p53 and down-regulation of the Bmi-1 oncogene; delayed neural cell differentiation; and impaired hippocampal synaptic transmission and plasticity. These findings provide *in vivo* experimental evidence that the NLS and C-terminus of PTHrP are essential not only for the regulation of neural cell proliferation and differentiation, but also for the maintenance of normal neuronal synaptic transmission and plasticity.

## Introduction

Parathyroid hormone-related peptide (PTHrP) was first identified in cancers associated with hypercalcemia [1]. PTHrP can signal through the parathyroid hormone type 1 (PTH)/PTHrP receptor (PTHR1), a G protein coupled receptor, which is important in skeleton and mammary gland development [2]. Normally PTHrP circulates at very low levels in the blood, but is produced in many cells and tissues, including those in the central nervous system (CNS), and plays a number of physiological roles through its paracrine/autocrine actions [3], [4]. PTHrP is a polyhormone which can be translated and processed into many smaller bioactive forms, including an N-terminal peptide, midregion, nuclear localization sequence (NLS) and C-terminal region [3]. The N-terminal region of PTHrP, i.e., PTHrP (1–36), mediates the “classical” PTH-like effect. The midregion, i.e., PTHrP (37–86), is involved in placental calcium transportation [5], and PTHrP (108–139), is believed to inhibit bone resorption. The NLS is located in PTHrP (87–107) and can translocate heterologous plasma proteins into the nucleolus [6]. Functionally, intranuclear PTHrP has been shown to increase cell proliferation and to delay apoptosis *in vitro*. Thus, endogenous PTHrP with its intact NLS, stimulates proliferation, for example, of vascular smooth muscle cells *in vitro*, via intranuclear targets that inactivate retinoblastoma protein (Rb), the inhibitory G1/S checkpoint regulator, by stimulating its hyperphosphorylation [7]. To examine the functional consequences of nuclear localization of PTHrP *in vivo*, we generated *Pthrp* knockin (KI) mice that express PTHrP (1–84), a truncated form of PTHrP that is missing the NLS and the C-terminal region but can still signal through its cell surface receptor [8]. *Pthrp* KI mice exhibit retarded growth and early senescence, leading to a rapid demise after birth [8]. These effects were accompanied by increased p16 and p21 levels and inhibition of cyclin E/Cdk2 and cyclin D1/Cdk4/Cdk6 activities, leading to cell cycle arrest in the G1 phase. Mice expressing PTHrP with deleted NLS/C-terminal domains also had lower nuclear levels of Bmi-1, a protein which promotes cell proliferation and suppresses genes that induce cellular senescence and cell death [9]. Together, these studies underline the importance of intracrine PTHrP action *in vitro* and *in vivo*.

In the CNS, PTHrP and PTHR1 are widely expressed especially in cerebral cortex, hippocampus and cerebellum [10], [11], [12], [13]. Brains of PTHrP^−/−^ mice are more susceptible to kainic acid neurotoxicity damage [14]. PTHrP was induced in reactive astrocytes of inflamed brain possibly as an inflammatory mediator in the CNS [15]. These studies suggest that PTHrP plays a protective role in the brain. In our previous study, we have reported that brains of *Pthrp* KI mice were both smaller and edematous and found a profound decrease in PCNA positive stem/progenitor cells in the subventricular zone and the hippocampus at E18.5 [8]. However, it is unclear whether the NLS and C terminus of PTHrP play a critical role in brain development and function by stimulating the proliferation and differentiation of neural stem cells and in maintaining normal neuronal synaptic transmission and plasticity. To answer this question, the phenotypes including brain shape and structures, the proliferation and differentiation of neural stem cells and hippocampal synaptic transmission and plasticity of *Pthrp* KI mice were compared to their wild-type littermates by using histology, immunohistochemistry, histomorphometry, Western blots and electrophysiological recordings.

## Materials and Methods

### Animals

Generation and characterization of the *Pthrp* KI and *Pthrp* knockout mice were preformed as our previously described [8], [16]. The genotype was confirmed as previously described [8], [16]. Wild-type littermates were used as controls in all the experiments. Animals were housed under standard conditions in the Medical Experimental Animal Center of Nanjing Medical University. All animal experiments were approved by the Animal Committee of Nanjing Medical University (Approval ID 2008-00318).

### Histology

Embryonic day 18.5 (E18.5), postnatal day 1 (P1), day 7 (P7) and day 14 (P14) *Pthrp* KI mice and their wild-type littermates, and E18.5 *Pthrp^−/−^* (*Pthrp* KO) mice and their wild-type littermates were anesthetized with inhaled ether and weighed on an electronic balance. The mice were then perfused by the intracardiac route with 5 ml cold saline followed by 10 ml 4% paraformaldehude in 0.1M phosphate buffer. Brains were removed, weighed and dissected into two symmetrical halves along the cerebral longitudinal fissure, and then post-fixed overnight at 4°C. The fixed brains were dehydrated, embedded in paraffin and 5 µm sagittal sections were cut on a rotary microtome. Sections were stained with hematoxylin and eosin (HE), or processed by terminal-deoxynucleotide transferase mediated d-UTP nick end labeling (TUNEL) or for immunohistochemistry as described below.

### Immunohistochemical and TUNEL staining

Sections were dewaxed, rehydrated and boiled in 0.01M PBS pH 7.4 for 20 minutes to retrieve antigen. Preimmune serum was substituted for the primary antibody as a negative control. After three 10 minute washes with PBS, sections were incubated with primary antibodies at 4°C overnight. Monoclonal mouse anti-proliferating cell nuclear antigen (PCNA) (1∶400, Dako, Denmark), anti-neuronal nuclei (NeuN) (1∶500, Millipore, Billereca, MS, USA), rat anti-myelin basic protein (MBP) (1∶200, Millipore,Billereca, MS, USA), and polyclonal rabbit anti-glial fibrillary acidic protein (GFAP) (1∶400, Dako, Copenhagen, Denmark) were used in this study. The sections were washed three times to remove unconjugated primary antibodies, and then the corresponding secondary antibodies were applied and incubated at room temperature for 45 minutes. The sections were then washed and incubated with the Vectastain Elite ABC Kit (Vector Laboratories, USA) for 30 minutes and diaminobenzidine (Vector laboratories,Burlingame, CA, USA) was used as a peroxidase substrate to yield a brown product. After washing with distilled water, the sections were counterstained with hematoxylin and coverslipped with neutral gum as mounting media. For TUNEL staining, sections were immersed in 200 ml 0.1 M citrate buffer, pH 6.0 and irradiated at 750 W (high power) microwave for 1 min and tested with the in situ Cell Death Detection Kit (Roche Diagnostics, Basel, Switzerland) according to the manual. Diaminobenzidine was used as substrate and hematoxylin counterstain was applied.

### Histomorphometric analysis

The sections were digitally photographed using an Olympus BX51 microscope equipped with a DP70 camera. Images were analyzed by Northern Eclipse software (Empix Imaging, Inc., NY, USA). All image analysis was based on a high power field (40×obj.) except for immunostaining for MBP. For the hippocampus cell density measurement, HE stained series sections were recorded on high power field and the hippocampus cell density was measured using Northern Eclipse image analysis software. The following parameters were used: cell numbers, positive cells, positive area and summary total gray (STG). STG = mean gray density of positive structures×positive area. The percentage of positive cells (%) = positive cell number/total cell number×100. The positive cell area (%) = positive area/field area×100.

### Neurosphere assay

Neurosphere assay was performed as described previously [17]. Briefly, the walls of the lateral ventricles were removed from 2-day-old wild-type and Pthrp KI mice and enzymatically dissociated in Hank's balanced saline solution (HBSS) buffer (5 mM KCl, 124 mM NaCl, 3.2 mM MgCl2, 100 lM CaCl2, 26 mM NaHCO3, and 10 mM glucose) containing 1 mg/ml trypsin at 37°C for 10 minutes, and mechanically dissociated. The tissue was centrifuged at 750 rpm for 5 minutes in soybean trypsin inhibitor. The dispersed cells were resuspended in HBSS buffer containing 0.7 mg/ml RNase free DNase and enzymatically disaggregated for 5 minutes at room temperature. The dissociated cells were centrifuged, resuspended in DMEM/F-12 containing 10 ng/ml epidermal growth factor, 10 ng/ml basic basic fibroblast growth factor, 1% B27, 1% N2, 2 mM glutamine and antibiotics (100 units/ml penicillin and 100 g/ml streptomycin). Cells were plated at 10 cells/µL in 24-well (0.5 mL/well) uncoated plates. The neurospheres were allowed to develop in a 95% air-5% CO_2_ humidified atmosphere at 37°C. The total number of spheres that formed in each well was counted at 3, 6 and 9 days and colonies 10–30 µm, 31–50 and >50 µm in diameter were counted respectively at 9 days.

### Western Blot

Proteins were extracted from the P14 hippocampus and quantitated on a nucleic acid and protein determinator (SmartSpec Plus Spectrophotometer, Eppendorf, Germany). Twenty microgram proteins of each sample were electrophoretically separated on SDS gel and transferred to nitrocellulose membranes. The membranes were incubated overnight at 4°C with polyclonal rabbit anti-PTHrP1–34 (1∶1000, Santa Cruz, CA, USA), Monoclonal mouse anti-Bmi-1 (1∶400, Millipore, Billerica, MS, USA), polyclonal rabbit anti-p16 (1∶1000, Santa Cruz, CA, USA), anti-p21 (1∶1000, Santa Cruz, CA, USA), monoclonal mouse anti-p53 (1∶1000, Santa Cruz, CA, USA), anti-p27 (1∶1000, Sigma, St. Louis, MO, USA), respectively, monoclonal mouse anti-β-tubulin (1∶2000, Santa Cruz, CA, USA) was used as a loading control. The goat anti-rabbit IgG-HRP (1∶2000, KPL, Gaithersburg, MD, USA) or goat anti-mouse IgG-HRP (1∶2000, KPL, Gaithersburg, MD, USA) corresponding to primary antibody were incubated 2 hours at room temperature. All the immunoreactive blots were detected with Chemiluminescent Substrate Kit and quantitated by Image J software.

### Electrophysiological recordings

Hippocampal slices were prepared from P14 *Pthrp* KI and their wild-type littermates as described previously [18]. In brief, the mice were anesthetized with ether and decapitated, and hippocampus was removed from brain. Coronal brain slices were cut into 350 µm thickness using a vibratome in ice-cold artificial cerebral spinal fluid (ACSF) containing 126 mM NaCl, 2.5 mM KCl, 1 mM MgCl_2_, and 20 mM glucose. ACSF was bubbled continuously with carbogen (95%O_2_/5%CO_2_) to maintain pH 7.4. Fresh slices were incubated with carbogenated ACSF at 34°C for at least 1.5 h before recording. For excitatory postsynaptic currents (EPSCs) recording, the hippocampal slices were transferred to a recording chamber, and CA1 pyramidal neurons were located under an upright microscope. The recording chamber (1.5 ml) was perfused at a rate of 4 ml/min, with an external recording solution which contained 119 mM NaCl, 26 mM NaHCO_3_, 2.5 mM KCl, 1 mM NaH_2_PO_4_, 1.3 mM MgCl_2_, 4 mM CaCl_2_, and 25 mM glucose, bubbled with 95% O_2_ and 5% CO_2_. Spontaneous α-amino-3-hydroxy-5-methyl-4-isoxazolepropionic acid receptors (AMPAR) and N-methyl-D-aspartate receptors (NMDAR)-mediated EPSCs of CA1 pyramidal neurons were evoked by stimulating the Schaffer fibers through a constant-current pulse delivered by a bipolar tungsten electrode and recorded with Axopatch-200B amplifier (Molecular Devices, USA). All the above recordings were conducted in low-Mg^2+^ (0.25 mM) ACSF. A stimulating electrode was placed in CA1 stratum radiatum at least 60–80 µm away from the cell body layer. The current intensity of test stimuli (25–50 µA) was set to produce half-maximal EPSPs. The baseline was recorded at least 10 min to ensure the stability of the response. Long-term potentiation (LTP) was induced with 200 synaptic stimuli at 2 Hz during a 2.5 min depolarization to 0 mV [18]. Data were collected with pClamp 9.2 and analyzed using Clampfit 9.2 software (Molecular Devices).

### Data analysis

All data were expressed as means ± SEM. Two groups comparison was analyzed by *Student's* t test and multiple group comparison was performed by two-way analysis of variance (ANOVA) using GraphPad software (GraphPad Software Inc., San Diego, CA, USA). A *p*-value less than 0.05 were considered statistically significant.

## Results

### PTHrP NLS and C terminus deficiency leads to abnormal brain shape and structures

**Figure 1:**
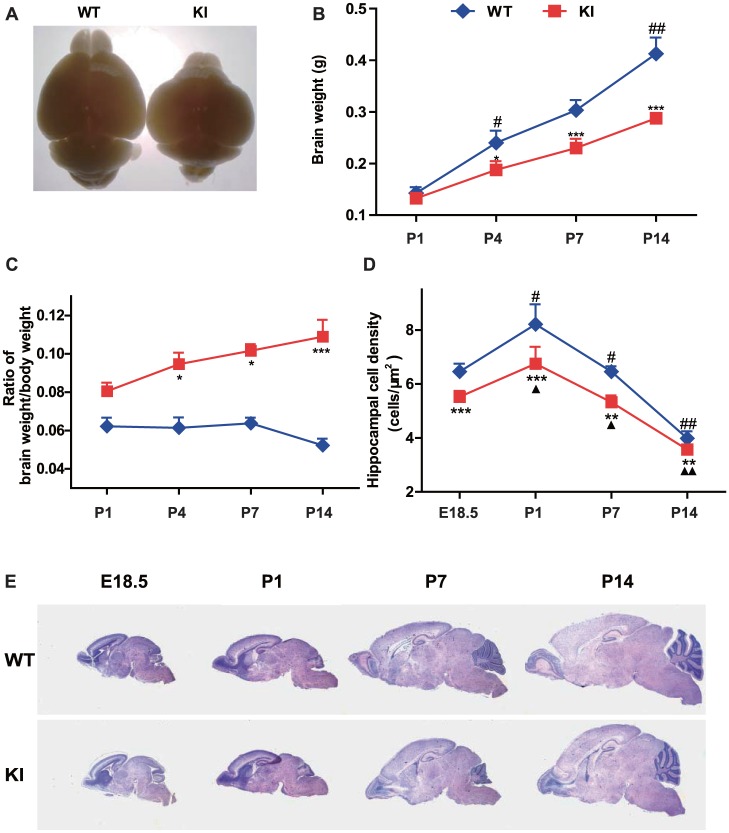
PTHrP NLS and C terminus deficiency leads to abnormal brain shape and structures. (A) Representative graphs of brains from postnatal day 14 (P14) wild-type (WT) and *Pthrp* KI mice. Measurements on postnatal day 1 (P1), day 4 (P4), day 7 (P7) and day 14 (P14) in wild-type (WT) and *Pthrp* KI mice of (B) the brain weight, (C) the ratios of brain weight to body weight, and (D) the hippocampal cell density. (E) Representative micrographs from WT and *Pthrp* KI mice of HE stained sagittal sections of brains from embryonic day 18.5 (E18.5), P1, P7 and P14 (magnification, ×12.5). Each value is the mean±SEM of determinations in 5 mice of each group. *, *P*<0.05; **, *P*<0.01; ***, *P*<0.001 in *Pthrp* KI mice relative to wild-type littermates. **#**, *P*<0.05; **##**, *P*<0.01 at the time point relative to the prior observed time point in WT mice. **▴**, *P*<0.05; **▴▴**, *P*<0.01 at the time point relative to the prior observed time point in *Pthrp* KI mice.

To evaluate the effect of PTHrP NLS and C terminus deficiency on brain morphogenesis, brain shape, weight and structures were compared between *Pthrp* KI mice and their wild-type littermates. The overall brain size and weight were reduced in *Pthrp* KI mice from embryonic day 18.5 (E18.5) to postnatal day 14 (P14) (Figs. 1A and B). The ratios of brain weight to body weight gradually increased however from P1 to P14 in P*thrp* KI mice and were significantly higher than their wild-type littermates from P4 to P14 (Fig. 1C). However, the hippocampus cell density was significantly reduced in P*thrp* KI mice compared to their wild-type littermates (Fig. 1D). Furthermore, at P14 (Fig. 1E), *Pthrp* KI mice had a shorter whole brain rostro-caudal axis but greater dorsal-ventral axis, smaller olfactory bulb, dramatic reduction of the thickness of the frontal cerebral cortex, and atrophy of the cerebellum at P7 and P14 (Figs. 1A and E). These results demonstrated that PTHrP NLS and C terminus deficiency resulted in abnormalities of brain shape and structures.

### PTHrP NLS and C terminus deficiency decrease neural cell proliferation and increase neural cell apoptosis

**Figure 2:**
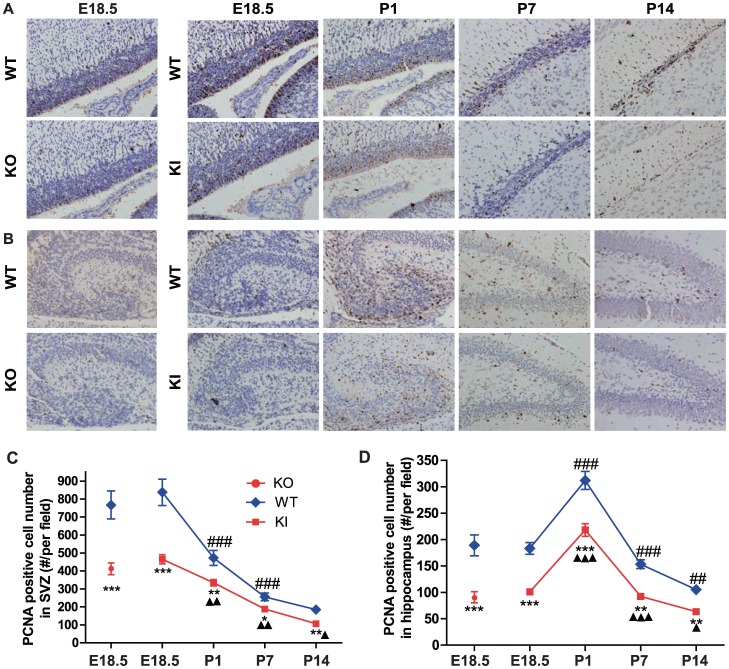
PTHrP NLS and C terminus deficiency results in a reduction of neural stem cells. Representative micrographs of sections in (A) the subventricular zone (SVZ) and (B) the hippocampus (HP) from the brains of E18.5 WT and P*thrp^−/−^* (KO) mice and E18.5, P1, P7 and P14 WT and *Pthrp* KI mice immunostained for PCNA (brown color, magnification, ×400). PCNA positive cell numbers in (C) SVZ and (D) HP (number/per field). Each value is the mean±SEM of determinations in 5 mice of each group. *, *P*<0.05; **, *P*<0.01; ***, *P*<0.001 in *Pthrp* KI mice relative to wild-type littermates. **##**, *P*<0.01; **###**, *P*<0.001 at the time point relative to the prior observed time point in WT mice. **▴**, *P*<0.05; **▴▴**, *P*<0.01; **▴▴▴**, *P*<0.001 at the time point relative to the prior observed time point in *Pthrp* KI mice.

**Figure 3:**
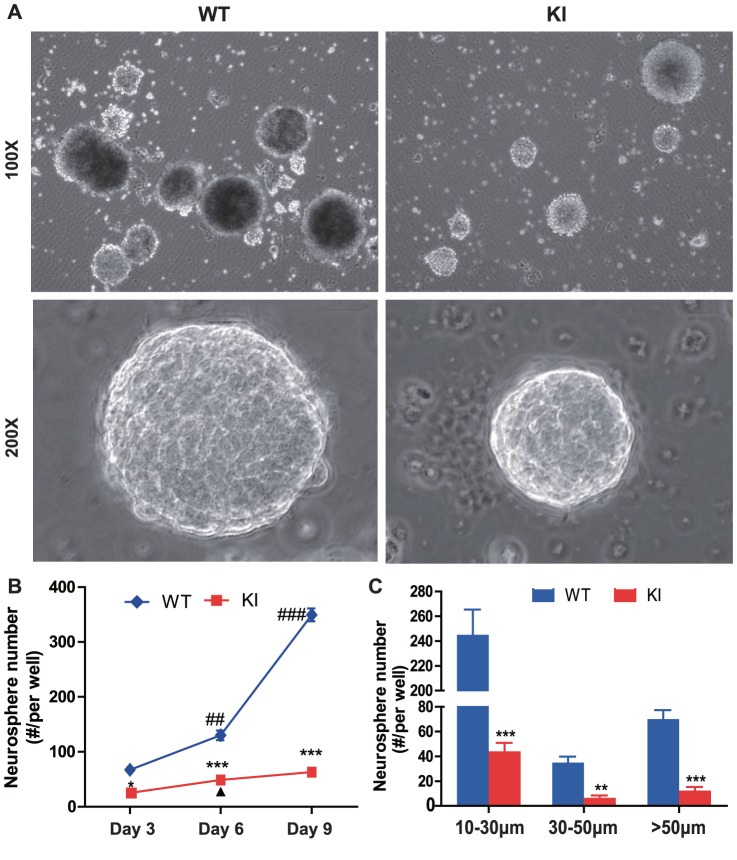
PTHrP NLS and C terminus deficiency results in a reduction of neurosphere forming efficiency. (A) Representative micrographs of neurospheres developed from the neurosphere assay of WT and KI mice. (B) Neurosphere numbers per well developed from 3, 6 and 9 day cultures. (C) Neurosphere numbers per well at different sizes developed from 9 day cultures. *, *P*<0.05; **, *P*<0.01; ***, *P*<0.001 in *Pthrp* KI mice relative to wild-type littermates. **##**, *P*<0.01; **###**, *P*<0.001 at the time point relative to the prior observed time point in WT mice. **▴**, *P*<0.05 at the time point relative to the prior observed time point in *Pthrp* KI mice.

**Figure 4:**
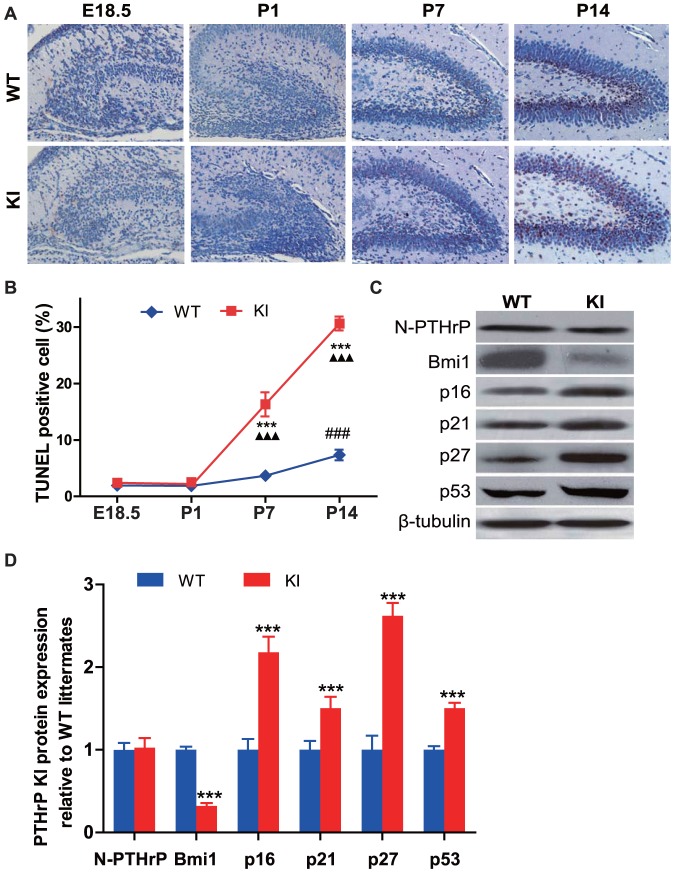
PTHrP NLS and C terminus deficiency increases neural cell apoptosis and up-regulate expression levels of CDKIs in the hippocampus. (A) Representative micrographs of sections from the hippocampus of brains from E18.5, P1, P7 and P14 WT and KI mice stained for apoptotic cells using the TUNEL technique (brown, magnification, ×400). (B) The percentage of apoptotic cells in hippocampus was quantified by computer-assisted image analysis. (C) Western blot analysis of hippocampus protein extracts for N-terminal PTHrP (N-PTHrP), Bmi1, p16, p21, p27 and p53. β-tubulin was used as loading control. (D) N-PTHrP, Bmi1, p16, p21, p27 and p53 protein levels relative to β-tubulin protein level were assessed by densitometric analysis and expressed relative to levels of WT mice. Each value is the mean±SEM of determinations in 5 mice of each group. ***, *P*<0.001 in *Pthrp* KI mice relative to wild-type littermates. **###**, *P*<0.001 at the time point relative to the prior observed time point in WT mice. **▴▴▴**, *P*<0.001 at the time point relative to the prior observed time point in *Pthrp* KI mice.

The proliferation of neural cells was examined by immunohistochemistry for proliferating cell nuclear antigen (PCNA). The results showed that PCNA positive cells were detected mainly in the subventricular zone (SVZ) and hippocampus. PCNA positive cells were maximal at E18.5 and decreased gradually from P1 to P14 in the SVZ, however, PCNA positive cells were maximal at P1 and decreased from P7 to P14 in the hippocampus (Figs. 2A and B). PCNA positive cells were decreased significantly in both SVZ and hippocampus at each time point in *Pthrp* KI mice compared to their wild-type littermates (Fig. 2). To assess the contribution to decreased neural cell proliferation of the PTHrP NLS and C terminus relative to the intact PTHrP molecule, the proliferation of neural cells was compared in *Pthrp* KI mice and *Pthrp* KO mice. Results showed that PCNA positive cells were decreased significantly in both the SVZ and hippocampus at E18.5 in *Pthrp* KO mice compared to their wild-type littermates, however, the decrease was not significantly different compared to E18.5 *Pthrp* KI mice (Fig. 2). We used the *in vitro* neurosphere assay as an index of the number of neural stem cells *in vivo*. Results showed that neurosphere numbers were reduced significantly in 3, 6 and 9 day cultures of *Pthrp* KI mice compared to their wild-type littermates and neurosphere numbers at 10–30 µm, 31–50 µm and >50 µm in diameter were also reduced significantly in 9 days cultures of *Pthrp* KI mice compared to their wild-type littermates (Fig. 3). Apoptosis of neural cells was detected by TUNEL assay. Results revealed that the percentage of apoptotic neural cells in dentate gyrus was not altered at E18.5 and P1 but increased significantly at P7 and P14 in *Pthrp* KI mice (Figs. 4A and B).

In view of the fact that decreased cell proliferation and increased cell apoptosis are often mediated by upregulated expression of cyclin dependent kinase inhibitors (CDKIs) [19], [20], we examined p16, p21, p27 and p53 protein expression as well as protein expression of the oncogene Bmi-1, a member of the Polycomb/trithorax group (Pc-G/trx-G) proteins [9], [21] in the P14 hippocampus. The expression of p16,p21, p27 and p53 were all up-regulated,while Bmi-1 was down-regulated in *Pthrp* KI mice compared with their WT littermates (Figs. 4C and D). However, the expression levels of N-terminual PTHrP were similar between *Pthrp* KI and their wild-type littermates as demonstrated using antibody against PTHrP1–34 (Figs. 4C and D). These results indicated that PTHrP NLS and C terminus deficiency caused the inhibition of neural stem cell proliferation and the induction of neural cell apoptosis by upregulating expression of CDKIs and downregulating expression of Bmi-1.

### PTHrP NLS and C terminus deficiency result in a delay of neural cell differentiation

**Figure 5:**
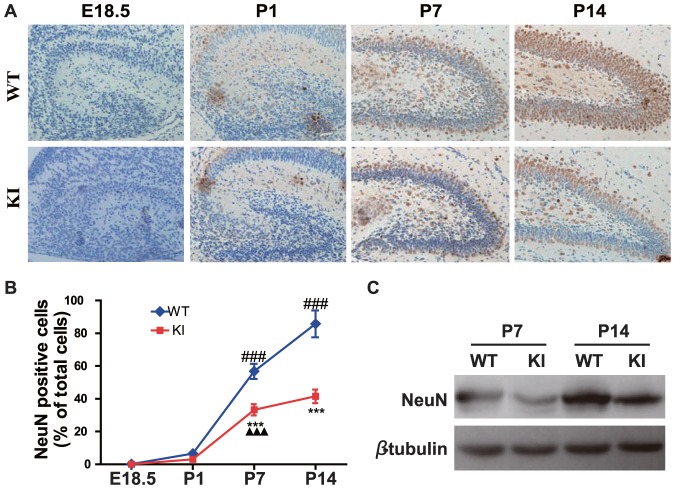
PTHrP NLS and C terminus deficiency results in a delay of neural stem cell differentiation into neurons. (A) Representative micrographs of sections from the hippocampus of brains of E18.5, P1, P7 and P14 WT and *Pthrp* KI mice immunostained for NeuN (brown color, magnification, ×400). (B) The percentage of NeuN positive cell number relative to total cell number in the hippocampus. (C) Western blot analysis of hippocampus protein extracts for NeuN. β-tubulin was used as loading control. Each value is the mean±SEM of determinations in 5 mice of each group. ***, P<0.001 in *Pthrp* KI mice relative to wild-type littermates. **###**, *P*<0.001 at the time point relative to the prior observed time point in WT mice. **▴▴▴**, *P*<0.001 at the time point relative to the prior observed time point in *Pthrp* KI mice.

**Figure 6:**
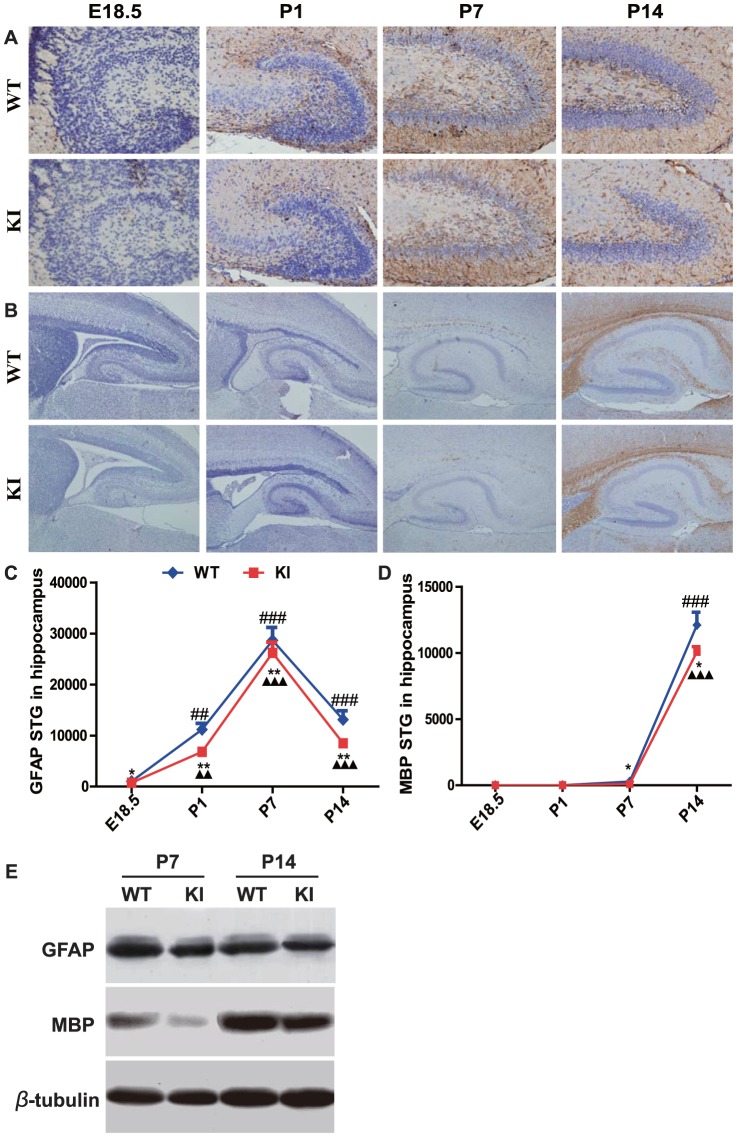
PTHrP NLS and C terminus deficiency results in a delay of neural stem cell differentiation into astrocytes and oligodendrocytes. Representative micrographs of sections from hippocampus of brains of E18.5, P1, P7 and P14 WT and *Pthrp* KI mice immunostained for (A) GFAP (brown color, magnification, ×400) and (B) MBP (brown color, magnification ×100). (C) The summary total gray (STG) of GFAP positive structures. (D) The STG of MBP positive structures. (E) Western blot analysis of hippocampus protein extracts for GFAP and MBP. β-tubulin was used as loading control. Each value is the mean±SEM of determinations in 5 mice of each group. *, *P*<0.05; **, *P*<0.01; ***, *P*<0.001 in *Pthrp* KI mice relative to wild-type littermates. **##**, *P*<0.01; **###**, *P*<0.001 at the time point relative to the prior observed time point in WT mice. **▴▴**, *P*<0.01; **▴▴▴**, *P*<0.001 at the time point relative to the prior observed time point in *Pthrp* KI mice.

To determine whether the defective brain development in *Pthrp* KI mice was associated with alterations of neural cell differentiation, the neural cell differentiation was examined by immunohistochemistry for NeuN, a neuron-specific nuclear protein, GFAP, an astrocyte specific marker, and MBP, an oligodendrocyte specific marker. The results showed that a few NeuN positive cells were detected in the dentate gyrus at E18.5 and P1 wild-type mice and more NeuN positive cells were detected in the outer granular layer and hilum of the dentate gyrus at P7 and P14 in wild-type mice (Fig. 5A). The granular layer of the dentate gyrus was thinner and the percentage of NeuN positive cells relative to total cells in the dentate gyrus was reduced significantly at P7 and P14 in *Pthrp* KI mice relative to their wild-type littermates (Figs. 5A and B). We also examined NeuN expression in the P7 and P14 hippocampus by Western blot. Consistent with alterations of the percentage of NeuN positive cells, the expression levels of NeuN were down-regulated in the P7 and P14 hippocampus from *Pthrp* KI mice (Fig. 5C). GFAP positive structures started to appear at E18.5 and gradually increased after birth (Fig. 6A). GFAP positive summary total gray (STG) was reduced significantly in the dentate gyrus at E18.5, P1, P7 and P14 in *Pthrp* KI mice compared to their wild-type littermates (Figs. 6A and C). The MBP positive structures were not detected at E18.5 and P1 brain, but appeared in the cingulate gyrus of P7 brains. In P14 wild-type brains, the MBP positive fibres arose from the molecular layer of the hippocampus fissure to form the tangential fibers which extended from the molecular layer, stratum lacunosum, stratum radiatum, stratum lucidum, pyramidal cell layer, stratum oriens and alveus to the corpus callosum. In *Pthrp* KI brain, the MBP positive tangential fibers were substantially reduced (Fig. 6B and C). MBP STG was also reduced significantly in the hippocampus at P7 and P14 *Pthrp* KI mice compared with their WT littermates (Fig. 6D). We also examined GFAP and MBP expression in the P7 and P14 hippocampus by Western blot. Consistent with immunohistochemical alterations of GFAP and MBP, the protein expression levels of GFAP and MBP by Western blot were down-regulated in the P7 and P14 hippocampus from *Pthrp* KI mice (Fig. 6E). Taken together, these results from immunohistochemistry and Western blots indicate that PTHrP NLS and C terminus deficiency result in a delay of neural stem cell differentiation into neurons, astrocytes and oligodendrocytes.

### PTHrP NLS and C terminus deficiency impair hippocampal synaptic transmission and plasticity

**Figure 7:**
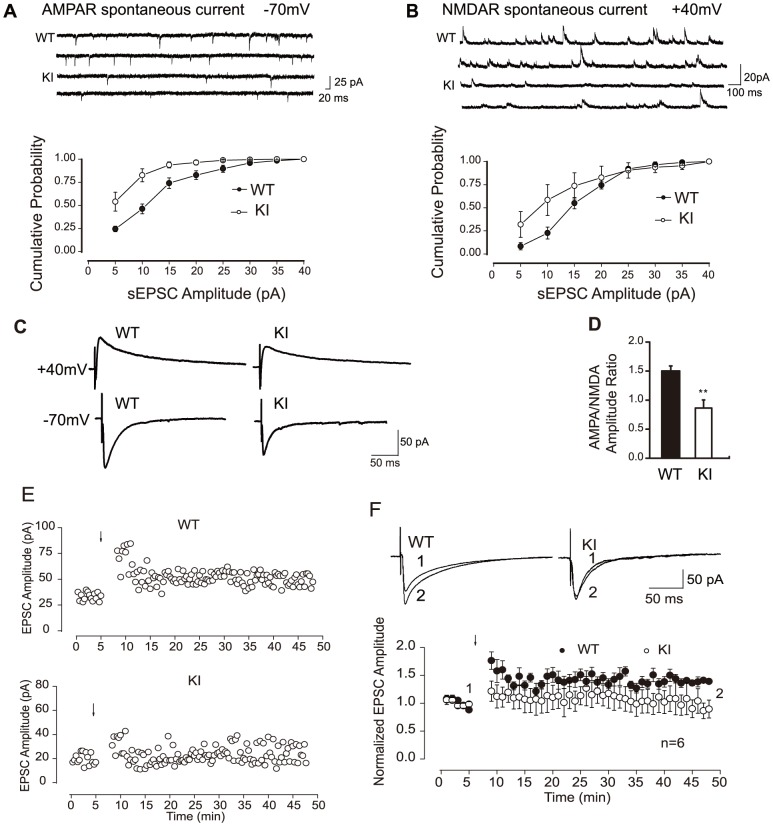
PTHrP NLS and C terminus deficiency impairs hippocampal synaptic transmission and plasticity. (A) Attenuation of spontaneous AMPA receptor-mediated postsynaptic currents (AMPA EPSCs). CA1 neurons were recorded at a −70 mV holding potential in conventional whole-cell patch mode. Top: Representative traces from CA1 neurons of P14 WT and *Pthrp* KI hippocampus. Both the frequency and amplitude of AMPA EPSCs were decreased in *Pthrp* KI mice. Bottom: Cumulative distribution of AMPA EPSCs amplitude (mean±SE) in WT and *Pthrp* KI mice. (B) Attenuation of spontaneous NMDA receptor-mediated postsynaptic currents (NMDA EPSCs). Spontaneous NMDA EPSCs were recorded at +40 mV in whole-cell mode. Top: Representative traces from neurons of WT and *Pthrp* KI hippocampus. Both the frequency and amplitude of NMDA EPSCs were decreased in *Pthrp* KI mice. Bottom: Cumulative distribution of NMDA EPSCs amplitude in P14 WT and *Pthrp* KI mice. (C) Representative traces display evoked AMPA EPSCs and NMDA EPSCs when cells were held at −70 mV and +40 mV, respectively. (D) Summary of statistical results shows the attenuation of the AMPA/NMDA ratio in *Pthrp* KI mice. (E) Sample experiments display LTP recorded in hippocampal CA1 neurons in P14 WT and KI mice. (F) Summary data show impared LTP in *Pthrp* KI mice. Compared with WT control, the magnitude of LTP in *Pthrp* KI mice was significantly attenuated. LTP was produced with a pairing protocol. Each value is the mean±SEM of determinations in 5 mice of each group. ** *P*<0.01, compared to wild-type littermates.

To assess whether the PTHrP NLS and C terminus deficiency impair hippocampal synaptic transmission and plasticity, we performed conventional whole-cell recordings in CA1 neurons in hippocampal slices prepared from P14 postnatal mice. Our results showed that the frequency and amplitude of both spontaneous AMPA receptor- and NMDA receptor-mediated excitatory postsynaptic currents (EPSCs) were decreased in *Pthrp* KI mice (Figs. 7A–B). In addition, the ratio of evoked AMPA receptor- and NMDA receptor-mediated synaptic responses (AMPA/NMDA) were also decreased significantly in *Pthrp* KI mice (Figs. 7C–D). To further investigate whether the synaptic plasticity was also impaired in *Pthrp* KI mice, we examined the long-term potentiation (LTP) induced with a pairing protocol. Our results revealed that the magnitude of LTP in *Pthrp* KI hippocampus was attenuated compared with wild-type control (Figs. 7E–F). Taken together, our data strongly suggest that the PTHrP NLS and C terminus deficiency impaired both synaptic transmission and plasticity in CA1 hippocampal neurons.

## Discussion

In this study, we employed a genetic approach to investigate the function of the PTHrP NLS and C terminus on brain development and function *in vivo*. Our results show that *Pthrp* KI mice displayed abnormal brain shape and structures, decreased neural cell proliferative capacity and increased apoptosis, delayed neural cell differentiation and impaired hippocampal synaptic transmission and plasticity. The decreased neural proliferation seen in both the SVZ and hippocampus in *Pthrp* KI mice was also observed in *Pthrp* KO mice suggesting that a similar phenotype occurs when the intact molecule is ablated. Although comparison of the brain phenotype in the PTHR knockout mice would also have been of interest, these animals die at embryonic day E11.5–E12.5, likely of heart disease [22] and it was therefore not possible to do comparative studies with this model. Our overall results are therefore compatible with a distinct phenotype caused by ablation of the PTHrP NLS and C-terminus.

In previous studies we found a profound decrease in PCNA positive stem/progenitor cells in the subventricular zone and the hippocampus in brains from E18.5 *Pthrp* KI mice [8]. In the current study, we not only confirmed our previous results from E18.5 *Pthrp* KI mice, but also found that PCNA positive stem/progenitor cells were highly concentrated in the subventricular zone at E18.5, especially at postnatal day 1 in the hippocampus in both wild-type and *Pthrp* KI mice. PCNA positive stem/progenitor cells were reduced significantly at all time points observed from E18.5 to postnatal day 14 in *Pthrp* KI mice. Previous reports have demonstrated that *in vitro*, deletion of PTHrP NLS inhibited the proliferation of smooth muscle cells [23], breast cancer cells [24] and prostate cancer cells [25] through an intracrine pathway. Our *in vivo* results indicate that PTHrP can stimulate the proliferation of neural stem/progenitor cells through the intracrine pathway. NLS-deleted PTHrP markedly inhibits arterial smooth muscle proliferation by coordinate up-regulation of p15 and p27 [23]. Decreased cellular proliferative capacity in multiple tissues including bone and bone marrow cells were associated with altered expression and subcellular distribution of the senescence associated tumor suppressor proteins p16, p21 and p27 and the oncogenes Cyclin D, pRb, and Bmi-1 in PTHrP NLS and C terminus deficient mice [8]. In this study, we also found that decreased proliferation of neural stem/progenitor cells was associated with the up-regulation of p16, p21, p27 and p53. These results suggest that PTHrP NLS can stmulate the proliferation of neural stem cells and inhibit apoptosis of neural cells by inhibiting cyclin-dependent kinase inhibitors (CDKIs) including p16, p21, p27 and p53.

Others have demonstrated that one of the major Bmi-1 targets is the *Ink4a/Arf* locus [9] that encodes a cyclin-dependent kinase inhibitor, p16, and a tumor suppressor, p19. In *Bmi1*-deficient mice, the expression of *Ink4a* and *Arf* is markedly increased in hematopoietic cells, and the enforced expression of *Ink4a* and *Arf* in hematopoietic stem cells resulted in cell cycle arrest and p53-dependent apoptosis, respectively [21]. In NSCs, particularly those derived from the SVZ and hippocampus, Bmi-1 is necessary for normal NSC self-renewal and survival, because it silences genes that encode the cell cycle inhibitors p16, p19, and p21 [26], [27], [28]. We have shown here that the expression of Bmi-1 is down-regulated significantly, whereas the expression of p16, p21, p27 and p53 is up-regulated significantly in hippocampus from *Pthrp* KI mice. These results suggest that PTHrP plays an intracrine role mediated via Bmi-1 to inhibit CDKIs.

Previous reports showed that treatment with PTHrP 1–141, but not PTHrP 1–86 for 24-hrs, resulted in a concentration-dependent increase in alkaline phosphatase in MC3T3-E1 pre-osteoblasts, suggesting that the NLS and C-terminus of PTHrP are responsible for PTHrP 1–141 stimulating osteoblast differentiation [29]. However, it was unclear what effect the NLS and C-terminus of PTHrP might have on neural cell differentiation. Another study demonstrated that Bmi-1 is not only required for their clonogenic self-renewal of I-type neuroblastoma cells, but also regulates their lineage-specific differentiation in a concentration dependent manner [30]. In this study, we examined the effect of PTHrP NLS and C terminus deficiency on neural cell differentiation *in vivo*. The differentiation of neurons, astroglial cells and oligodendrocytes were delayed in the *Pthrp* KI mice,suggesting that the NLS and C-terminus of PTHrP may enhance the NSC differentiation into neurons, astroglial cells and oligodendrocytes. Further studies will be required to delineate the importance of these actions in regulation of neural cell differentiation.

The hippocampus plays an important role in spatial navigation, learning and memory and cognitive functions [31]. EPSCs are mediated by both NMDAR and AMPAR [32], which are postsynaptic glutamate receptors and involved in the synaptic transmission in the hippocampus [33]. We found that the NLS and C-terminus of PTHrP are required not only for the regulation of neural cell proliferation and differentiation, but also for maintaining normal neuronal synaptic transmission and LTP. These effects in *Pthrp* KI mice might be due to the reduction in the number of synapses during electrophysiological testing. In view of the fact that mature neurons, as indicated by NeuN staining, were fewer, there might be fewer synapses to participate in the evoked-synaptic responses. We also showed that LTP is compromised by the deficiency of PTHrP NLS and C terminus. The MBP immunostaining results indicate that the schaffer collateral fibers [34], which is an important pathway for long-term potentiation (LTP), were dramatically affected by the deletion of PTHrP NLS and C terminus, suggesting that schaffer collateral fibers were not well developed or were less functional.

This study therefore is the first to provide *in vivo* experimental evidence that the NLS and C-terminus of PTHrP is essential for brain development and neuronal synaptic transmission and plasticity by stimulating neural cell proliferation and differentiation and inhibiting neural cell apoptosis associated with the regulation of cyclin dependent kinase inhibitors p16, p21, p27 and p53 and the oncogene Bmi-1.
